# Participation in community groups increases the likelihood of PrEP awareness: New Orleans NHBS-MSM Cycle, 2014

**DOI:** 10.1371/journal.pone.0213022

**Published:** 2019-03-12

**Authors:** Yusuf Ransome, Meagan Zarwell, William T. Robinson

**Affiliations:** 1 Department of Social and Behavioral Sciences, Yale School of Public Health, New Haven, Connecticut, United States of America; 2 Center for AIDS Intervention Research, Medical College of Wisconsin, Milwaukee, Wisconsin, United States of America; 3 Louisiana Office of Public Health, STD/HIV Program, and Department of Behavioral and Community Health Sciences, LSU School of Public Health, New Orleans, Louisiana, United States of America; Hunter College, UNITED STATES

## Abstract

**Background:**

Gay, bisexual, and other men who have sex with men (GBM) have the highest proportion of incident HIV infection. Pre-exposure prophylaxis (PrEP) use and screening for sexually transmitted infections (STIs) are primary HIV prevention strategies, however, uptake remains low. Social capital, collective resources generated through social connections, are associated with lower HIV risk and infection. We investigated social capital in association with PrEP indicators among GBM.

**Methods:**

Analyses included (N = 376) GBM from the 2014 National HIV Behavioral Surveillance (NHBS) in New Orleans. Multiple regression methods assessed the association between one item within each of eight domains from the Onyx and Bullen Social Capital Scale and: awareness and willingness to use PrEP. Analyses are adjusted for age, race, education, sexual intercourse with women, and health insurance.

**Results:**

Forty percent of GBM were 18–29 years, 52 percent White. Sixty percent were willing to use PrEP. Social capital was above 50 percent across 7 of 8 indicators. Community group participation (vs no participation) was associated with higher likelihoods of PrEP awareness (adjusted Prevalence Ratio [aPR] = 1.41, 95% Confidence Interval [CI] = 1.02, 1.95). None of the seven remaining social capital indicators were significantly associated with any of the PrEP outcomes.

**Conclusions:**

Community groups and organizations could be targeted for interventions to increase uptake of HIV prevention strategies among GBM in New Orleans

## Introduction

Seventy percent of new HIV infections in the United States (US) occur among gay, bisexual, and other men who have sex with men (GBM) [1]. An estimated one in six (16.7 percent) of GBM will become infected with HIV in their lifetime [2]. New HIV diagnosis rates among GBM are highest within the southern states [3]. For instance, the estimated diagnosed HIV prevalence was twice as high in Louisiana compared to the national rate [4]. In New Orleans, Louisiana—the setting of this current study—the primary risk factor for new HIV diagnosis is reported being GBM, which accounted for 56 percent of infections in 2016, among persons who reported a risk factor [5].

Individual factors that increase risk of HIV acquisition and transmission for GBM include unprotected receptive anal intercourse [6]. Social network factors that increase risk of HIV acquisition and transmission for GBM include higher HIV prevalence within smaller, dense sexual networks [7, 8]. Combination approaches that include biomedical HIV-prevention modalities such as pre-exposure prophylaxis (PrEP), and consistent condom use are recommended to reduce HIV transmission, especially among GBM [9, 10]. PrEP is an oral antiretroviral (ARV) medication taken daily by HIV-negative individuals who are at high social or behavioral risk [11]. Studies demonstrated that PrEP has have high efficacy in reducing HIV acquisition among GBM, when taken consistently [12].

Despite the importance of PrEP for reducing HIV incidence among GBM, uptake of this technology remains suboptimal among this group. Two independent national studies estimated that about 4 percent of GBM were using PrEP [13, 14]. Low uptake of PrEP and related prevention strategies among GBM are largely attributed to social and structural risk determinants [15] including financial hardship, lack of social support, and HIV stigma and conspiracy beliefs [16].

Psychosocial factors, such as internal and external homonegativity [17], internal social anxiety and discrimination-directed abuse also influence sexual behavior and possibly uptake of HIV prevention and care among GBM. For instance, one study showed that social anxiety, activated by interpersonal fears of rejection among GBM led to avoiding safe-sex negotiations, which in-turn was associated with unprotected anal intercourse [18]. External factors such as verbal, and childhood physical abuse directed towards GBM have also shown to impact high risk sexual behavior directly and indirectly through higher risk of syndemics psychosocial problems (e.g., depression and heavy alcohol use) [19].

While we know much about the individual psychosocial determinants that influence HIV prevention behaviors, only recently has there been work documenting the role of interpersonal factors such as perceived social norms and how norms facilitate the uptake of HIV prevention strategies for GBM [20]. Beyond inter and intrapersonal factors, experts have called for more research to investigate social-ecological or community-level determinants of biomedical HIV prevention [21] such as PrEP outcomes [22], in population-based settings. However, one systematic review published in 2018 reported that no studies investigated associations between societal and community-level factors that facilitate PrEP acceptability and uptake [23].

Social capital is one social-ecological factor, defined broadly as collective resources generated through social connections [24, 25] that can facilitate individual behavior change and health. There are two primary theoretical approaches or traditions within social capital and health research that influence which indicators are used in studies. First, social cohesion is the most widely used approach/tradition [26], which focuses on the cognitive aspects of social capital such as perceptions of trust, norms of reciprocity, and participation in one’s network or community [27]. Measures typically assess one’s level of participating in community-related events, one’s ability to obtain information, and one’s degree of feelings of safety and trust in their neighborhood [27]. Next is the structural forms of social capital, which focuses on the availability of organizational resources, patterns of civic engagement in neighborhoods, and informal social control among a collective unit [28, 29]. Second, social networks is another theoretical approach/tradition of social capital, which focuses on the resources embedded within network ties [24, 30]. Measures from the social network perspective includes the resource generator, which tallies the actual resources available in one’s network [31]. There is currently no consensus about how to best measure social capital, and there is significant variation on how the concept is applied in HIV research. One recent systematic review that summarized the association between social capital and HIV/AIDS outcomes in the US found that some studies conceptualized social capital as an individual-level attribute while other studies conceptualized it as a property of the community/collective [32]. The systematic review found a wide range of survey instruments used to assess social capital, and documented that very few studies incorporated measures from both social capital approach/traditions [32].

Despite theoretical and methodological differences across prior studies, social capital has been documented to primarily have protective associations with health outcomes [33]. Specific to HIV-related outcomes, social capital has been associated with lower HIV risk behaviors [34], lower HIV diagnosis [35] and HIV incidence rates [36], higher adherence of ARV therapy [37], and suppressed viral load [38]. One quantitative study among GBM in Swaziland found that higher social capital participation was associated with 30 percent higher likelihood of testing for HIV in the past 12 months [39]. One study among GBM in the US found that psychosocial strengths, which included social support and social capital as a composite variable was associated with lower risk of condomless anal intercourse [40]. Regarding uptake of HIV prevention, one study among GBM in South Africa documented that strengthening social capital links between community-based HIV prevention volunteers and GBM is important to mitigate barriers such as mistrust and community homonegativity [41].

One pathway through which social capital facilitates an increase likelihood of HIV prevention and reduce HIV risk is buffering mental health outcomes related to social stigma and homonegativity [42]. For instance, one quantitative study among young black HIV positive GBM in the US, among those who report depressive symptoms, lower social capital was associated with 27 percent lower odds of viral suppression [43]. Within the US, emerging work suggests that social relations developed within group membership shape GBM’s sexual risk behaviors [44]. The current evidence from prior studies and emerging studies suggests that social capital may be important in increasing PrEP uptake among GBM.

However, we could not identify any studies in the US that assessed this association. We therefore investigated whether social capital indicators, from the social cohesion approach/tradition, were associated with awareness of PrEP and willingness to use PrEP among GBM in the US [32]. We included PrEP awareness as an outcome because diffusion of innovation is a key pathway through which social capital is associated with health [45]. Our study data were collected in 2014, less than two years after the Food and Drug Administration approved Truvada for PrEP. Next, PrEP research is still in its infancy and enhancing awareness is the second step in the PrEP care continuum [46]. Last, PrEP awareness and willingness to use PrEP are independent indicators for actual uptake [47].

Social capital theory and the breadth of findings from prior empirical work reveal that social capital indicators, particularly those based on the social cohesion perspective, can be protective of HIV outcomes. We hypothesized then, that GBM who report social cohesion aspects such as participation in community groups and sense of trust in their community will have higher likelihoods of being aware of PrEP and of being willing to use PrEP.

## Methods

### Ethical approval

“All procedures performed in studies involving human participants were in accordance with the ethical standards of the institutional and/or national research committee and with the 1964 Helsinki declaration and its later amendments or comparable ethical standards.” The ethics committee Institutional Review Board (IRB) at Louisiana State University Health Sciences Center and Louisiana Department of Health approved the study.

### Participants

A total of 553 GBM were surveyed during the New Orleans CDC National HIV Behavioral Surveillance (NHBS) study in 2014. Eligibility requirements included cisgender men, 18 years of age or older, who were residents of the New Orleans metropolitan area, able to take the survey in English, and reported ever having oral or anal sex with another man. Participants with complete data on age, race, education, insurance status, ever had sex with a woman, and the social capital variables were N = 493, and then we excluded HIV positive GMB, resulting in a final analytic sample size of N = 376 HIV negative GBM. Informed consent was obtained from all participants to complete the anonymous survey and HIV test. All procedures were approved by the Louisiana State University Health Sciences Center and Louisiana Department of Health’s Institutional Review Board (IRB).

### Recruitment

Participants were recruited through venue-based time-space sampling (VBTS) as specified within the NHBS protocol. While details of the NHBS sampling protocol have been described elsewhere [48], VBTS employs a two-stage sampling design using a monthly calendar to schedule specific days and times for recruitment events at venues such as bars, sex clubs, and dance clubs. Between July and December 2014, a total of 79 recruitment events were held in venues frequented by GBM in New Orleans. At recruitment events, GBM who crossed a threshold or line of recruitment were systematically approached by members of our team and screened for eligibility. Eligible men were asked to participate in completing an anonymous survey and offered the option of HIV testing. Participants were reimbursed a $25 cash-value gift card for completing the survey and an additional $25 cash-value gift card for completing an HIV test. Information about prevention services in New Orleans, and counseling materials were provided to all study participants. Verbal consent was provided by all participants before they initiated the survey.

### Measures

The NHBS contains a core instrument that covers a wide spectrum of questions on sociodemographics, sexual behavior, substance use, HIV and STI testing, and PrEP. A locally developed questionnaire included questions about social capital adapted from the Onyx and Bullen Social Capital Scale [27].

#### Sociodemographics

Age group (1 = 18–29, 2 = 30–39, 3 = 40 and older); race (1 = Black and Other vs 0 = non-Hispanic White); education (1 = less than high school, 2 = some college, 3 = college degree or higher); and currently have health insurance (1 = yes vs 0 = no). We also included a variable sex with a woman ever (1 = yes vs 0 = no) because of prior associations with sexual risk behavior [49], we thought it was plausible that this behavior may influence awareness and willingness to use PrEP.

#### Social capital

The items in our social capital scale are based on the social cohesion approach/tradition, which includes indicators such as trust, participation in community events, and neighborhood connections [50]. Respondents were asked a series of eight questions—one question within each of eight domains—modified from the Onyx and Bullen Social Capital Scale [27]. Domains and questions were: (*Value of Life*: Do you feel valued by society?; *Work Connections*: Are your co-workers or classmates also your friends?; *Tolerance of Diversity*: Do you enjoy living among people of different lifestyles?; *Community Group Participation*: Are you an active member of a local organization or club?; *Social Agency*: If you need information to make a life decision, do you know where to find that information?; *Trust/Safety*: Do you feel safe walking down your street after dark?; *Neighborhood Connections*: Have you visited a neighbor in the past week? *Friend Communication*: In the past week, how many times did you communicate with friends using your phone?). As a continuous measure, friend communication was not significantly related to any of the outcomes. The choice to dichotomize this measure using the median value of 40 was made due to extreme outliers. Thus, a “1” response indicated speaking to friends more than 40 times per week whereas a “0” indicated communicating less than 40 times per week. Responses to all dichotomous questions were (1 = yes vs 0 = no/don’t know/refused).

#### PrEP awareness and willingness

Awareness of PrEP was ascertained from the question, “Before today, have you ever heard of people who do not have HIV taking antiretroviral medicines, to keep from getting HIV?” Willingness to take PrEP was ascertained from the question, “Would you be willing to take anti-HIV medicines every day to lower your chances of getting HIV?” Responses to the PrEP questions were (1 = yes vs 0 = no/don’t know).

### Analysis plan

Frequency distributions were calculated for all sociodemographic, social capital, and PrEP variables. Adjusted prevalence ratios (aPR) were calculated through Log-Poisson regression, which estimated the aPRs for the PrEP variables with two categories: (1 = yes and 0 = no/don’t know = reference). Each model included relevant covariates age, race, education, sex with women (ever), and current health insurance. In all models, the eight social capital variables were included in one block because they were not highly or significantly correlated. All analyses are conducted among the HIV-negative sample since those eligible for PrEP cannot be HIV-positive.

## Results

All the study results are reported in Table 1. Approximately 41 percent of GBM sampled were between the ages of 18–29 years, and 52 percent were white. Forty-seven percent were aware of PrEP and 60 percent were willing to use PrEP. Frequency of those reporting yes to seven of the eight social capital indicators was greater than 50 percent. Community group participation was the lowest reported social capital indicator, where 26 percent said yes.

**Table 1:** Description of the sample and multivariable association among predictors and PrEP awareness and willingness. New Orleans National HIV Behavioral Surveillance (NHBS)-MSM Cycle, 2014.

Total, n (376)	N (%)	Aware of PrEP (N = 369)	Willing to use PrEP (N = 351)
		aPR, 95% CI	aPR, 95% CI
Age group, 18–29, ref	153 (41%)	1	1
Age group, 30–39	93 (25%)	1.14 (0.77, 1.66), *p* = 0.50	1.00 (0.72, 1.41), *p* = 0.96
Age group, 40 and older	130 (35%)	0.83 (0.55, 1.23), *p* = 0.354	0.61 (0.42, 0.91), *p* = 0.01
Race, Non-Hispanic White (ref)	209 (57%)	1	1
Race, Black and other	167 (43%)	0.72 (0.51, 1.01), *p* = 0.06	0.99 (0.73, 1.33), *p* = 0.94
Sex with women (ever), No (ref)	162 (43%)	1	1
Sex with women (ever) Yes	214 (57%)	1.05 (0.77, 1.44), *p* = 0.74	0.89 (0.67, 1.18), *p* = 0.42
Education, Less than high school (ref)	85 (23%)	1	1
Education, Some college	97 (26%)	1.72 (0.93, 3.17), *p* = 0.08	1.27 (0.84, 1.93), *p* = 0.26
Education, College degree or higher	194 (52%)	2.92 (1.66, 5.11), *p* = 0.00	1.26 (0.85, 1.88), *p* = 0.25
Current health insurance, No (ref)	104 (28%)	1	1
Current health insurance, Yes	272 (72%)	0.77 (0.53, 1.10), *p* = 0.16	0.78 (0.46, 1.32), *p* = 0.62
Community group participation, yes	98 (26%)	1.41 (1.02, 1.95), *p* = 0.04	1.20 (0.88, 1.64), *p* = 0.26
Social agency, yes	332 (88%)	1.29 (0.72, 2.33), *p* = 0.38	0.87 (0.57, 1.32), *p* = 0.52
Trust and safety, yes	291 (77%)	1.06 (0.73, 1.54), *p* = 0.76	0.95 (0.66, 1.33), *p* = 0.76
Neighborhood connections, yes	239 (64%)	1.13 (0.82, 1.57), *p* = 0.45	0.98 (0.74, 1.31), *p* = 0.92
Value of life, yes	309 (82%)	0.93 (0.61, 1.45), *p* = 0.77	1.05 (0.71, 1.55), *p* = 0.80
Work connections, yes	290 (77%)	0.97 (0.67, 1.41), *p* = 0.87	0.88 (0.63, 1.22), *p* = 0.45
Tolerance of diversity, yes	363 (97%)	0.95 (0.42, 2.17), *p* = 0.91	1.65 (0.65, 4.18), *p* = 0.29
Friend communication, yes	195 (52%)	1.00 (0.73, 1.38), *p* = 0.99	0.95 (0.70, 1.26), *p* = 0.66
^a^Aware of PrEP, yes	174 (47%)		
^a^Willing to use PrEP, yes	210 (60%)		

^a^ = In multivariable analysis, the PrEP variables are defined as (1 = yes vs 0 = no/don’t know, and 0 is the referent group)

aPR = Adjusted Prevalence Ratio, CI = Confidence Interval

### Social capital and PrEP

GBM participating (vs not participating) in community groups were 40 percent more likely to be aware of PrEP (aPR = 1.40, 95% CI = 1.02, 1.95, *p* = 0.04). No other social capital variables were significantly associated with willingness to use PrEP in this sample.

### Other variables and PrEP

Being 40 years of age or older (vs 18–29 years) was not significantly associated with lower likelihood of PrEP awareness (aPR = 0.83, 95% CI = 0.55, 1.23, *p* = 0.35) but was significantly associated with willingness to use PrEP (aPR = 0.61, 95% CI = 0.42, 0.91, *p* = 0.01). College degree or higher (vs less than high school) was significantly associated with higher likelihood of being aware (aPR = 2.92, 95% CI = 1.66, 5.11, *p* = 0.00) but not willingness to use PrEP (aPR = 1.26, 95% CI = 0.85, 1.88, *p* = 0.25). Race was not significantly associated with either PrEP outcome.

## Discussion

Social capital has been identified as an important social determinant for HIV prevention, especially among GBM globally [39], yet there is a paucity of work on the topic in the US [32]. Our study contributes to the literature by identifying what specific domains or indicators of social capital may be useful to intervene on among GBM. In our study, although 47 percent of GBM were aware of PrEP, 60 percent of GBM were willing to use PrEP, which is higher than estimates reported among international cohorts conducted around similar times (e.g., 48 percent among GBM in Scotland, UK) [51]. Specific to the US, our estimates are similar to those found in an NHBS sample of GBM from Philadelphia, PA—another urban city [52]. We found that GBM participating in community groups reported significantly higher likelihoods of being aware of PrEP, but the positive association did not reach statistical significance for willingness to use PrEP. These results point to a strong potential for improving uptake of PrEP within the community group settings where diffusion of PrEP awareness could lead to increased willingness and uptake of PrEP may occur among GBM. We expected awareness may be low among participants since PrEP was approved by the FDA less than two years from the time of the study, however we are encouraged by the high willingness to use PrEP.

Although the association in multivariable analysis was not significant, lack of significance could have been attributed to additional factors not measured here, including structural barriers such as healthcare access or stigma. Another potential reason for lack of significance could be specific mediating mechanisms, such as social group membership that is mediating participation and willingness to use PrEP [53]. Social group membership in constructed families has been identified as a potential GBM-specific measure of social capital [54], and empirical measures of this concept has been associated with sexual risk behaviors such as condomless anal sex [44]. Nevertheless, given that PrEP is now more widely accessible, advertised, and affordable (six years after FDA approval), a follow-up replication study is important to assess whether community group participation remains associated with willingness and actual PrEP uptake.

The importance of our study findings for policy and prevention is that these data suggest there is awareness of PrEP in the community groups where GBM participate. While we did not have qualitative data about the content or what occurs in the community groups, we think there could be an opportunity for the local Health Department to deliver interventions in these settings. Community groups could be a place to provide on-site consultation and screening for PrEP use, as well as mental and other behavioral support, and motivational interview trainings to improve uptake, retention in using PrEP, and high PrEP adherence. There is one recent successful model of social capital intervention to improve engagement in HIV care for GBM living with HIV in the US South [55] that could potentially be adapted to improve uptake of HIV prevention strategies.

The remaining social capital indicators were not statistically associated with any PrEP outcome. Based on the social cohesion perspective of social capital, [25] we might have expected social agency, and trust and safety in one’s neighborhood to be significant predictors of HIV preventions strategies for GBM. We anticipated this because there is often high external and community stigma and discrimination that GBM experience within their communities, which have been a barrier for accessing HIV prevention resources [56, 57].

There are some study limitations. NHBS survey items on PrEP were based on self-report and responses could be biased. However, NHBS responses have been shown to be reliable because the study sites used highly trained interviewers who have high rapport with participants [58, 59]. VBTS sampling is not a probability-based design and may miss GBM who do not attend the venues selected. Nevertheless, VBTS is currently a gold standard for recruiting multisite national samples of GBM [48].

Although there is no consensus on how best to assess social capital, we elected items with high factor loadings from each of eight domains derived from the modified Onyx and Bullen social capital scale. Next, we dichotomized responses into yes vs no/don’t know/refused, although the original measures are based on a 4-point Likert-type response scale [27]. Prior studies on the topic in the US that used the Onyx and Bullen scale differed widely in how they quantified social capital in association with HIV outcomes [60–62]. Therefore, we cannot assess the validity of our measurement approaches, and so we are unaware of how our methodological choices could have affected our results. Nevertheless, we had strict time constraints to assess multiple other items (including social capital) in the local questionnaire, so we incorporated short answer responses to maximize time.

## Conclusion

Despite those limitations, this was the first study to investigate social capital in association with PrEP outcomes in the US [63] and perhaps globally. The findings contribute to the prevailing theory that social capital can be a protective resource for HIV prevention, with participation in community groups being one mechanism that facilitates the association [36, 64, 65].

We recommend future work on this topic. As we discussed in the introduction, current social capital and health research has overwhelmingly focused on the social cohesion perspective that taps the cognitive aspects of social capital [26]. There are other perspectives such as structural social capital where indicators include density of civic and social organizations [28]. There is also the social network perspective, which considers the density and strength of ties in one’s networks. Thus, one other avenue for research is to investigate whether membership in endogenous social networks within the LGBT community in which GBM participate, including constructed families is associated with higher willingness and uptake of PrEP.

## References

[1] Centers for Disease Control and Prevention. HIV among gay and bisexual men Atlanta, GA: National Center for HIV/AIDS, VIral Hepatitis, STD, and TB Prevention; 2017 [cited 2018 May 3]. http://www.webcitation.org/query?url=https%3A%2F%2Fwww.cdc.gov%2Fhiv%2Fpdf%2Fgroup%2Fmsm%2Fcdc-hiv-msm.pdf&date=2018-05-03.

[2] Hess KL, Hu X, Lansky A, Mermin J, Hall HI. Lifetime risk of a diagnosis of HIV infection in the United States. Ann Epidemiol. 2017;27(4):238–43. 10.1016/j.annepidem.2017.02.003. 28325538 PMC5524204

[3] Villarosa L. America’s hidden HIV epidemic: why do America’s black gay and bisexual men have a higher HIV rate than any country in the world? New York, NY: The New York Times; 2017 [cited 2018 October 2].

[4] Rosenberg ES, Grey JA, Sanchez TH, Sullivan PS. Rates of prevalent HIV infection, prevalent diagnoses, and new diagnoses among men who have sex with men in US states, metropolitan statistical areas, and counties, 2012–2013. JMIR Public Health Surveill. 2016;2(1):e22 10.2196/publichealth.5684 27244769 PMC4887662

[5] Louisiana Department of Health. Louisiana HIV, AIDS, and early syphillis surveillance, fourth quarter report, Vol 14, No. 4 New Orleans, LA: Office of Public Health; 2017 [cited May 3 2018]. http://www.webcitation.org/query?url=http%3A%2F%2Fldh.la.gov%2Fassets%2Foph%2FHIVSTD%2FHIV_Syphilis_Quarterly_Reports%2F4thQuarter2017_HIV_Syphilis_Report.pdf&date=2018-05-03.

[6] Beyrer C, Baral SD, van Griensven F, Goodreau SM, Chariyalertsak S, Wirtz AL, et al Global epidemiology of HIV infection in men who have sex with men. Lancet. 2012;380(9839):367–77. 10.1016/S0140-6736(12)60821-6 22819660 PMC3805037

[7] Hermanstyne KA, Green HDJ, Cook R, Tieu H-V, Dyer TV, Hucks-Ortiz C, et al Social network support and decreased rsk of seroconversion in Black msm: results of the BROTHERS (HPTN 061) Study. J Acquir Immune Defic Synd. 2018;78(2):163–8. 10.1097/qai.0000000000001645 29424789 PMC5953785

[8] Eaton LA, Kalichman SC, Cherry C. Sexual partner selection and HIV risk reduction among black and white men who have sex with men. Am J Public Health. 2010;100(3):503–9. 10.2105/AJPH.2008.155903 . 20075328 PMC2820059

[9] Sullivan PS, Carballo-Diéguez A, Coates T, Goodreau SM, McGowan I, Sanders EJ, et al Successes and challenges of HIV prevention in men who have sex with men. Lancet. 2012;380(9839):388–99. 10.1016/S0140-6736(12)60955-6 22819659 PMC3670988

[10] Maulsby C, Jain K, Sifakis F, German D, Flynn CP, Holtgrave D. Individual-evel and partner-level predictors of newly diagnosed HIV infection among black and white men who have sex with men in Baltimore, MD. AIDS Behav. 2015;19(5):909–17. 10.1007/s10461-014-0861-5 25092514

[11] Centers for Disease Control and Prevention. Pre-Exposure Prophylaxis (PrEP) Atlanta, GA: CDC; 2017 [cited 2017 September 3]. http://www.webcitation.org/6tDRkl99I.

[12] Grant RM, Lama JR, Anderson PL, McMahan V, Liu AY, Vargas L, et al Preexposure chemoprophylaxis for HIV prevention in men who have sex with men. N Engl J Med. 2010;363(27):2587–99. 10.1056/NEJMoa1011205 . 21091279 PMC3079639

[13] Parsons JT, Rendina HJ, Lassiter JM, Whitfield THF, Starks TJ, Grov C. Uptake of HIV pre-exposure prophylaxis (PrEP) in a national cohort of gay and bisexual men in the United States: the Mmotivational PrEP cascade. J Acquir Immune Defic Synd. 2017;74(3):285–92. 10.1097/QAI.0000000000001251 28187084 PMC5315535

[14] Hoots BE, Finlayson T, Nerlander L, Paz-Bailey G, Group NHBSS, Wortley P, et al Willingness to take, use of, and indications for pre-exposure prophylaxis among men who have sex with men—20 US cities, 2014. Clin Infect Dis. 2016;63(5):672–7. 10.1093/cid/ciw367 27282710 PMC5009903

[15] Millett GA, Peterson JL, Flores SA, Hart TA, Jeffries WL 4th, Wilson PA, et al Comparisons of disparities and risks of HIV infection in black and other men who have sex with men in Canada, UK, and USA: a meta-analysis. Lancet. 2012;380(9839):341–8. 10.1016/S0140-6736(12)60899-X. 22819656

[16] Ayala G, Bingham T, Kim J, Wheeler DP, Millett GA. Modeling the impact of social discrimination and financial hardship on the sexual risk of HIV among latino and black men who have sex with men. Am J Public Health. 2012;102(S2):S242–S9. 10.2105/ajph.2011.300641 . 22401516 PMC3477911

[17] Garcia J, Parker C, Parker RG, Wilson PA, Philbin M, Hirsch JS. Psychosocial implications of homophobia and HIV stigma in social support networks: insights for high-impact HIV prevention among black men who have sex with men. Health Educ Behav. 2016;43(2):217–25. 10.1177/1090198115599398 27037286 PMC4973624

[18] Hart TA. Integrated cognitive behavioral therapy for social anxiety and HIV prevention for gay and bisexual men. Cogn Behav Pract. 21(2):149–60. 10.1016/j.cbpra.2013.07.001

[19] Tulloch TG, Rotondi NK, Ing S, Myers T, Calzavara LM, Loutfy MR, et al Retrospective reports of developmental stressors, syndemics, and their association with sexual risk outcomes among gay men. Arch Sex Behav. 2015;44(7):1879–89. 10.1007/s10508-015-0479-3 26089251 PMC4559573

[20] Schnarrs PW, Gordon D, Martin-Valenzuela R, Sunil T, Delgado AJ, Glidden D, et al Perceived social norms about oral PrEP use: differences between African–American, Latino and White gay, bisexual and other men who have sex with men in Texas. AIDS Behavior. 2018;Online first:1–15. 29603111 10.1007/s10461-018-2076-7 PMC10372781

[21] Kippax S, Holt M. The state of social and political science research related to HIV: a report for the International AIDS Society. South Wales, Australia: National Centre on HIV Social Research. The University of New South Wales, 2009.

[22] Amico KR, Stirratt MJ. Adherence to preexposure prophylaxis: current, emerging, and anticipated bases of evidence. Clin Infect Dis. 2014;59(suppl_1):S55–S60. 10.1093/cid/ciu266 24926036 PMC4060253

[23] Peng P, Su S, Fairley CK, Chu M, Jiang S, Zhuang X, et al A global estimate of the acceptability of Pre-exposure Prophylaxis for HIV among men who have sex with men: a systematic review and meta-analysis. AIDS Behav. 2018;22(4):1063–74. 10.1007/s10461-017-1675-z 28176168

[24] Lin N. Building a network theory of social capital. Connections. 1999;22(1):28–51.

[25] Kawachi I, Berkman L. Social cohesion, social capital and health In: Kawachi I, Berkman L, Glymour M, editors. Social Epidemiology, 2nd Edition New York, NY: Oxford University Press; 2014 p. 291–319.

[26] Kawachi I, Subramanian S. Social epidemiology for the 21st century. Soc Sci Med. 2017;196(January):240–5. 29113687 10.1016/j.socscimed.2017.10.034

[27] Onyx J, Bullen P. Measuring social capital in five communities. J Appl Behav Sci. 2000;36(1):23–42.

[28] Villalonga-Olives E, Kawachi I. The measurement of social capital. Gac Sanit. 2015;29(1):62–4. 10.1016/j.gaceta.2014.09.006 25444390

[29] Putnam R. Bowling alone: America’s declining social capital. J Democr. 1995;6(1):65–78.

[30] Bourdieu P, Wacquant L. An Invitation to Reflexive Sociology. Chicago, IL: University of Chigago Press; 1992.

[31] Van der Gaag M, Webber M. Measurement of individual social capital In: Kawachi I, Subramanian S, Kim D, editors. Social Capital and Health. New York, NY: Springer Science and Business Media; 2005 p. 29–50.

[32] Ransome Y, Thurber KA, Swen M, Crawford ND, German D, Dean LT. Social capital and HIV/AIDS in the United States: knowledge, gaps, & future directions. SSM-population health. 2018;5(August):73–85. 29892697 10.1016/j.ssmph.2018.05.007 PMC5991916

[33] Kawachi I, Subramanian SV, Kim D, editors. Social capital and health. New York, NY: Springer Science + Business Media LLC; 2008.

[34] Pronyk PM, Harpham T, Morison LA, Hargreaves JR, Kim JC, Phetla G, et al Is social capital associated with HIV risk in rural South Africa? Soc Sci Med. 2008;66(9):1999–2010. 10.1016/j.socscimed.2008.01.023 18299168

[35] Ransome Y, Galea S, Pabayo R, Kawachi I, Braunstein S, Nash D. Social capital is associated with late HIV diagnosis: an ecological analysis. J Acquir Immune Defic Synd. 2016a;73(2):213–21. 27632146 10.1097/QAI.0000000000001043 PMC5026389

[36] Frumence G, Killewo J, Kwesigabo G, Nyström L, Eriksson M, Emmelin M. Social capital and the decline in HIV transmission–A case study in three villages in the Kagera region of Tanzania. SAHARA J Soc Asp H. 2010;7(3):9–20. 10.1080/17290376.2010.9724964 21409300

[37] Phillips JC, Webel A, Rose CD, Corless IB, Sullivan KM, Voss J, et al Associations between the legal context of HIV, perceived social capital, and HIV antiretroviral adherence in North America. BMC Public Health. 2013;13:736 736 10.1186/1471-2458-13-736 23924399 PMC3750916

[38] Mukoswa GM. Association between social capital and HIV treatment outcomes in South Africa. Witwatersrand, Johannesburg: School of Public Health, Faculty of Health Sciences, University of the Witwatersrand, Johannesburg; 2015.

[39] Grover E, Grosso A, Ketende S, Kennedy C, Fonner V, Adams D, et al Social cohesion, social participation and HIV testing among men who have sex with men in Swaziland. AIDS Care. 2016;28(6):795–804. 10.1080/09540121.2015.1131971 . 26824888

[40] Hart TA, Noor SW, Adam BD, Vernon JRG, Brennan DJ, Gardner S, et al Number of psychosocial strengths predicts reduced HIV sexual risk behaviors above and beyond syndemic problems among gay and bisexual men. AIDS Behav. 2017;21(10):3035–46. 10.1007/s10461-016-1669-2 28050650

[41] Tucker A, de Swardt G, Struthers H, McIntyre J. Understanding the needs of township men who have sex with men (MSM) health outreach workers: exploring the interplay between volunteer training, social cpital and critical consciousness. AIDS Behav. 2013;17(1):33–42. 10.1007/s10461-012-0287-x 22903420

[42] Stahlman S, Grosso A, Ketende S, Sweitzer S, Mothopeng T, Taruberekera N, et al Depression and social stigma among MSM in Lesotho: implications for HIV and sexually transmitted infection prevention. AIDS Behav. 2015;19(8):1460–9. 10.1007/s10461-015-1094-y 25969182 PMC4858167

[43] Hussen SA, Easley KA, Smith JC, Shenvi N, Harper GW, Camacho-Gonzalez AF, et al Social capital, depressive symptoms, and HIV viral suppression among young black, gay, bisexual and other men who have sex with men living with HIV. AIDS Behav. 2018;Online First:1–9. 29619586 10.1007/s10461-018-2105-6 PMC6076871

[44] Zarwell MC, Robinson WT. The influence of constructed family membership on HIV risk behaviors among gay, bisexual, and other men who have sex with men in New Orleans. Journal of Urban Health. 2017;Online First:1–9. 29047021 10.1007/s11524-017-0203-9 PMC5906379

[45] Nguyen H, Shiu C, Peters C. The relationship between Vietnamese youths’ access to health information and positive social capital with their level of HIV knowledge: Results from a national survey. Vulnerable Child. 2015;10(1):67–78.

[46] Nunn AS, Brinkley-Rubinstein L, Oldenburg CE, Mayer KH, Mimiaga M, Patel R, et al Defining the HIV pre-exposure prophylaxis care continuum. AIDS (London, England). 2017;31(5):731. 10.1097/QAD.0000000000001385 PMC5333727 28060019

[47] Cohen SE, Vittinghoff E, Bacon O, Doblecki-Lewis S, Postle BS, Feaster DJ, et al High interest in pre-exposure prophylaxis among men who have sex with men at risk for HIV-infection: baseline data from the US PrEP demonstration project. J Acquir Immune Defic Synd. 2015;68(4):439. 10.1097/QAI.0000000000000479 PMC4334721 25501614

[48] MacKellar DA, Gallagher KM, Finlayson T, Sanchez T, Lansky A, Sullivan PS. Surveillance of HIV risk and prevention behaviors of men who have sex with men—a national application of venue-based, time-space sampling. Public Health Rep. 2007;122(1_suppl):39–47. 17354526 10.1177/00333549071220S107 PMC1804106

[49] Friedman MR, Wei C, Klem ML, Silvestre AJ, Markovic N, Stall R. HIV infection and sexual risk among men who have sex with men and women (MSMW): a systematic review and meta-analysis. PLoS ONE. 2014;9(1):e87139 10.1371/journal.pone.0087139 24498030 PMC3907399

[50] Kawachi I, Berkman L. Social cohesion, social capital and health. Social Epidemiology. 2000.

[51] Frankis J, Young I, Flowers P, McDaid L. Who will use pre-exposure prophylaxis (PrEP) and why?: understanding PrEP awareness and acceptability amongst men who have sex with men in the UK–a mixed methods study. PLOS ONE. 2016;11(4):e0151385 10.1371/journal.pone.0151385 27093430 PMC4836740

[52] Adams JW, Shinefeld J, Brady KA. Acceptability of oral preexposure prophylaxis among men who have sex with men in Philadelphia. J Acquir Immune Defic Synd. 2016;73(3):e62–e5. 10.1097/qai.0000000000001139 27454249

[53] Zarwell M, Ransome Y, Barak N, Gruber D, Robinson WT. PrEP Indicators, social capital, and social group memberships among gay, bisexual, and other men who have sex with men Cult Health Sex. 2019;In Press:e0208781 10.1080/13691058.2018.1563912. PMC6684860 30724712

[54] Zarwell M, Robinson WT. Development of a social capital scale for constructed families of gay, bisexual, and other men who have sex with men. PLOS ONE. 2018;13(12):e0208781 10.1371/journal.pone.0208781 30543653 PMC6292593

[55] Hussen SA, Jones M, Moore S, Hood J, Smith JC, Camacho-Gonzalez A, et al Brothers building brothers by breaking barriers: development of a resilience-building social capital intervention for young black gay and bisexual men living with HIV. AIDS Care. 2019;Online first:1–8. 10.1080/09540121.2018.1527007 30626207 PMC8344093

[56] Oldenburg CE, Perez-Brumer AG, Hatzenbuehler ML, Krakower D, Novak DS, Mimiaga MJ, et al State-level structural sexual stigma and HIV prevention in a national online sample of HIV-uninfected men who have sex with men in the United States. AIDS 2015;29(7):837–45. 10.1097/QAD.0000000000000622 25730508 PMC4439297

[57] Baral S, Holland CE, Shannon K, Logie C, Semugoma P, Sithole B, et al Enhancing benefits or increasing harms: community responses for HIV among men who have sex with men, transgender women, female sex workers, and people who inject drugs. J Acquir Immune Defic Synd. 2014;66(Suppl):S319–S28. 25007203 10.1097/QAI.0000000000000233

[58] Lansky A, Sullivan PS, Gallagher KM, Fleming PL. HIV behavioral surveillance in the US: a conceptual framework. Public Health Rep. 2007a;122(1_suppl):16–23. 17354523 10.1177/00333549071220S104 PMC1804114

[59] Lansky A, Drake A, DiNenno E, Lee C-W. HIV behavioral surveillance among the US general population. Public Health Rep. 2007b;122(1_suppl):24–31. 10.1177/00333549071220S105 PMC1804108 17354524

[60] Nokes K, Johnson MO, Webel A, Rose CD, Phillips JC, Sullivan K, et al Focus on increasing treatment self-efficacy to improve Human Immunodeficiency Virus treatment adherence. J Nurs Scholarsh. 2012;44(4):403–10. 10.1111/j.1547-5069.2012.01476.x 23121723 PMC3593227

[61] Webel AR, Cuca Y, Okonsky JG, Asher AK, Kaihura A, Salata RA. The impact of social context on self-management in women living with HIV. Soc Sci Med. 2013;87(Supplement C):147–54. 23631790 10.1016/j.socscimed.2013.03.037 PMC3656470

[62] Webel AR, Wantland D, Rose CD, Kemppainen J, Holzemer WL, Chen W-T, et al A cross-sectional relationship between social capital, self-compassion, and perceived HIV symptoms. J Pain Symptom Manage. 2015;50(1):59–68. 10.1016/j.jpainsymman.2014.12.013 . 25659523 PMC4492802

[63] Ransome Y, Dean LT, editors. Research on social capital and HIV in the United States: results, challenges and future directions 21st International AIDS Conference (AIDS 2016); 2016; Duban, South Africa: International AIDS Society.

[64] Campbell C, Scott K, Nhamo M, Nyamukapa C, Madanhire C, Skovdal M, et al Social capital and HIV competent communities: The role of community groups in managing HIV/AIDS in rural Zimbabwe. AIDS Care. 2013;25(Suppl 1):S114–S22. 10.1080/09540121.2012.748170 23745625 PMC3701935

[65] Gregson S, Terceira N, Mushati P, Nyamukapa C, Campbell C. Community group participation: can it help young women to avoid HIV? an exploratory study of social capital and school education in rural Zimbabwe. Soc Sci Med. 2004;58(11):2119–32. 10.1016/j.socscimed.2003.09.001 15047071

